# Perceived stress as a risk factor of unemployment: a register-based cohort study

**DOI:** 10.1186/s12889-018-5618-z

**Published:** 2018-06-13

**Authors:** Maiken Holm Mæhlisen, Alexander Arndt Pasgaard, Rikke Nørmark Mortensen, Henrik Vardinghus-Nielsen, Christian Torp-Pedersen, Henrik Bøggild

**Affiliations:** 1 Public Health and Epidemiology Group, Department of Health Science and Technology, Aalborg University, Niels Jernes Vej 14, 9220 Aalborg Øst, Denmark; 2 Unit of Epidemiology and Biostatistics, Aalborg University Hospital, Sdr. Skovvej 15, 9000 Aalborg, Denmark

**Keywords:** Unemployment, Psychological stress, Cohen’s perceived stress scale, Perceived stress, Socioeconomic status

## Abstract

**Background:**

Although unemployment and high levels of perceived stress have been associated in cross-sectional studies, the direction of causation is unknown. We prospectively examined if high levels of perceived everyday life stress increased the risk of subsequent unemployment and further if differences existed between socioeconomic status-groups.

**Methods:**

We included 9335 18–64-year-old employed respondents of a health survey (North Denmark Health Profile 2010) in which Cohen’s Perceived Stress Scale was used to assess the level of perceived stress. Data were linked individually to national administrative registers. Cox proportional hazards model was used to investigate the association between perceived stress quintiles and risk of unemployment during 98 weeks of follow-up. Analyses were further performed in subgroups defined by education and income.

**Results:**

In total, 224 people (10.4%) of the high stress group became unemployed during follow-up, which was higher than the lower stress groups. After adjusting for gender, age, education and income, the risk of unemployment was 1.64 (95% CI: 1.28;2.11) in the high stress group compared to the low stress group. After adjusting for gender and age, a similar trend was observed across different education levels and among the lower income groups, but no higher risk of unemployment due to perceived stress was found among the higher income groups. However, there was no statistically significant interaction between perceived stress and income level (*p* = 0.841) or perceived stress and education level (*p* = 0.587).

**Conclusion:**

Perceived everyday life stress nearly doubled the risk of subsequent unemployment in a working population. No statistically significant interactions between SES and perceived stress were found. This indicates that stress prevention among the working population should not solely focus on stress in the workplace but also include stress from everyday life.

**Electronic supplementary material:**

The online version of this article (10.1186/s12889-018-5618-z) contains supplementary material, which is available to authorized users.

## Background

Unemployment has been associated with high levels of perceived stress in cross-sectional studies [1, 2], but in these studies it is not known if perceived stress is a risk factor of unemployment as the direction of causation is unknown.

Longitudinal studies have found that sickness absence [3], poor health [4–7] and poor health behavior [6–8] increased the risk of subsequent unemployment. Since these factors are potential consequences of perceived stress [9, 10], it is possible that perceived stress affects the risk of unemployment, which should be examined in a longitudinal study.

In cross-sectional studies, high levels of perceived stress have been found among people with low socioeconomic status (SES) measured by education and income level [11, 12]. These people have also been suggested to be more vulnerable to high levels of stress than high SES-groups [13]. This could indicate an increased risk of unemployment from perceived stress among people with low SES compared to those with high SES. Furthermore, previous follow-up studies have found that the risk of unemployment was higher among people with low SES compared to those with high SES [5, 14]. The influence of SES on the association between perceived stress and risk of unemployment seem complex and SES might either confound or modify the effect of perceived stress on unemployment.

Unemployment has been stated as a public health problem [15]. Research concerning potential risk factors of unemployment often focus on work conditions and work environment [15]. Knowledge of perceived everyday life stress as a potential risk factor of unemployment could be relevant for stress reduction among the working population. Furthermore, a possible deviating risk among SES-groups would help targeting high-risk groups.

The objective of this study was therefore to examine if high levels of perceived everyday life stress among a large cohort of employed people increased the risk of unemployment in a longitudinal study and further if differences existed between SES-groups.

## Methods

### Design and data sources

We conducted a cohort study with 98 weeks of follow-up (from 22nd March 2010 to 5th February 2012). Survey data from the North Denmark Health Profile 2010 [16] was individually linked with information from national administrative registers using the unique personal identification number of all Danish residents [17]. The North Denmark Health Profile 2010 included a Danish version of the 10-item Cohen’s Perceived Stress Scale (PSS) to assess the level of perceived stress [11]. Information on employment status was provided by the Danish Register for Evaluation of Marginalization (DREAM) [18]. The register contains weekly information on public transfer payments to all Danish citizens including unemployment benefits, sickness benefits, retirement pensions, state educational grants and maternity leaves [18]. The Population’s Education Register provided information on current and highest completed education authorised by the Danish Ministry of Education [19]. Self-reported information on education level and employment status [16] was used if information was missing in the registers. The Income Statistics Register, which is provided by the Danish Tax authorities, contains information on taxable income of Danish residents [20]. The Danish Civil Registration System provided information on age, gender and emigration [17]. The Danish Register of Causes of Death [21] was used to identify participants who died during follow-up.

### Study population

**Fig. 1:**
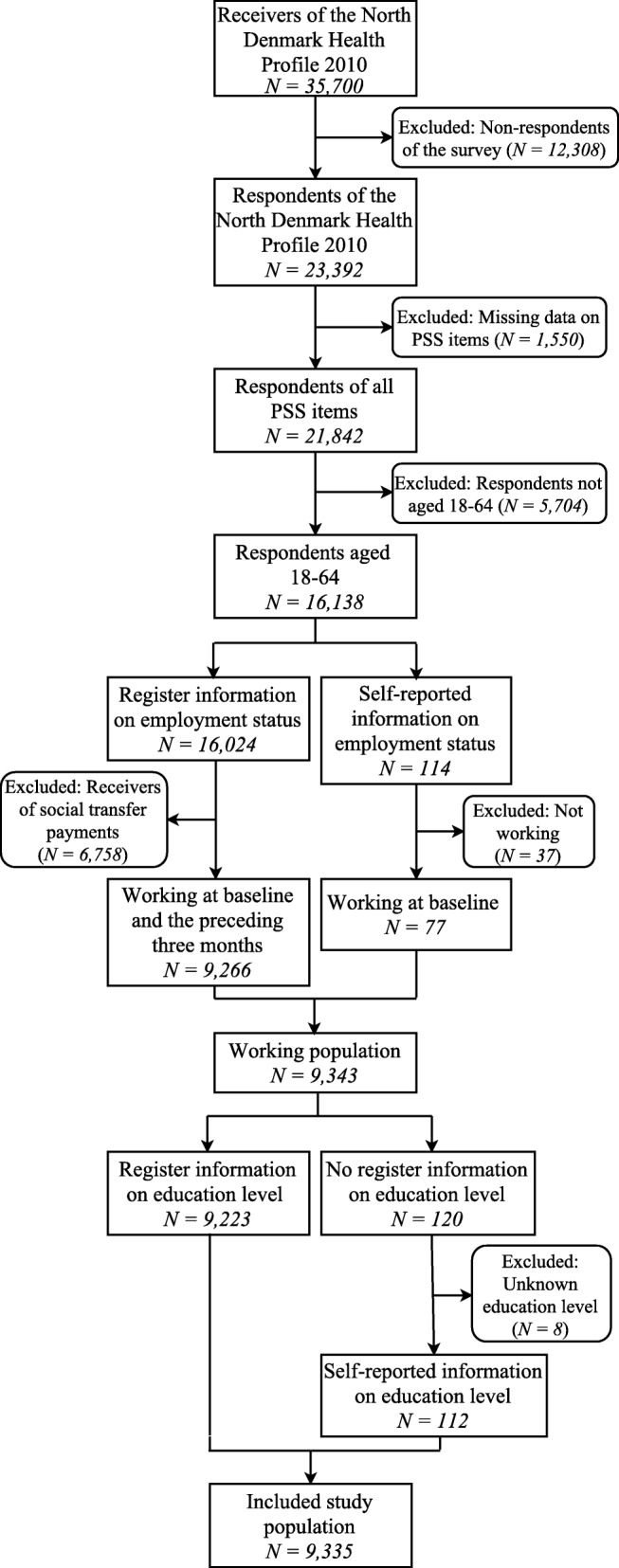
Flowchart of the included study population (*N = 9335*)

The study population was drawn from respondents of the North Denmark Health Profile 2010, which was sent to a random sample of 35,700 persons aged 16 years and above. The sample was stratified based on the 11 municipalities of the northern Region of Jutland, Denmark (570,000 inhabitants). 23,392 persons responded (response rate 65.5%) of which 21,842 persons answered all PSS items. Generally, the response rate was lowest among men. Furthermore, especially 16–34-year-old men and women did not respond [16]. We included respondents, who were 18–64 years old (*N = 16,138*), to represent a working population. At the time of the study, public retirement was possible at the age of 65. Only respondents, who were working at baseline and the preceding 3 months, were included to minimize the risk of including recently employed people, who might be on probation or very short-term temporary contracts. Employment status was assessed through DREAM (*N = 16,024*). Employed respondents were identified as citizens with no public transfer payments from 5th November 2009 to 22nd March 2010. Self-reported employment status [16] was used if people were not registered in DREAM (*N = 114*). Non-employed respondents (*N = 6795*) and respondents with missing information (*N = 8*) were excluded. This resulted in a study population of 9335 persons (Fig. 1).

### Perceived everyday life stress

The level of perceived stress was assessed through PSS, which measures the extent of unpredictability, lack of control and overload in the respondent’s life during the last month [11, 16]. The answers of each of the 10 items were scored from 0 to 4 ranging the five possible answers “*never”* to “*very often*”. The scores were added up ranging from 0 to 40 with a high score indicating a high level of perceived stress [11]. As the PSS do not have score cut-offs [22], the sum of the PSS-score was divided into quintiles for interpretation purpose.

### Unemployment

Identification of the first episode of unemployment during follow-up was based on the weekly transfer payments of two types of unemployment benefits; voluntary unemployment insurance benefits (DREAM-codes 111–113, 151–152, 211–299) and social security benefits (DREAM-codes 130–149) registered in DREAM (see groupings of transfer payments in Additional file 1: Table S1). These codes represent unemployed who are assessed ready to undertake work by the social office. In Denmark, receiving unemployment insurance benefits requires membership of an unemployment fund for at least 1 year prior to unemployment and a specific amount of education or work experience [23]. Those who do not meet these requirements are entitled to receive social security benefits although specific financial requirements have to be met [24].

### Sickness absence

First episode of sickness absence during follow-up was identified as receipt of sickness benefit registered in DREAM [18]. In DREAM, registration of sickness absence is not uniform. Only sickness absence > 21 days was registered unless the employee suffered from a certified chronic disease or the company was insured by which the employee would receive sickness benefit from the first day of sickness absence [25].

### Covariates

Age was grouped into 18–25, 26–54 and 55–64 years of age. Household income was calculated as the average of 2007, 2008 and 2009. If the respondent lived with a partner, the household income was divided by 1.5 to allow for sharing of resources [26]. Household income (Euro) was divided into quartiles; 1: < 44,763, 2: 44,763–55,978, 3: 55,979–67,524 and 4: > 67,524. Highest achieved education in 2009 was divided into three groups based on the International Standard Classification of Education (ISCED) [27]; basic (ISCED level 0–3; early childhood education, primary education, lower secondary education and general upper secondary education), vocational (ISCED level 3; vocational upper secondary education) and higher education (ISCED level 5–8; bachelor’s, master’s and doctor or equivalent level).

Information on lifestyle factors and self-rated health was self-reported [16]. Smoking status was categorized into three groups; never smoker, former smoker and current smoker. Body Mass Index (BMI) was calculated from information on height and weight [16] and divided into four groups based on WHO standards; underweight (BMI < 18.5), normal weight (18.5 ≤ BMI < 25), overweight (25 ≤ BMI < 30) and obesity (BMI ≥ 30). Alcohol consumption was based on information on units per day during the week [16]. One unit equals 12 g of pure alcohol*.* Alcohol consumption was dichotomized into low (< 180 g/week for women and < 264 g/week for men) and high (> 168 g/week for women and > 252 g/week for men) level based on recommendations on alcohol consumption from the Danish Health Authorities [28]. Self-rated health was assessed through the question: “*In general how do you assess your own health?*” [16] and dichotomized into good (“*Excellent*”/“*Very good*”/“*Good*”) and poor (“*Moderate*”/“*Poor*”).

### Statistical analyses

Descriptive statistics of baseline characteristics and outcome in the five stress quintiles were presented including outcome of non-respondents of the PSS. Cumulative incidence proportion of unemployment by perceived stress was estimated using the Aalen-Johansen estimator. Cox proportional hazards model was used to investigate the association between perceived stress quintiles at baseline and risk of unemployment during follow-up with lowest stress quintile as reference. To allow for the stratified sample design, the R-package ‘survey’ was used [29]. Unadjusted (model 1) and multivariate analyses including possible confounders (age, gender, SES) (model 2: fully adjusted) were performed. All analyses were further performed in subgroups defined by SES adjusting for gender and age.

To test the proportional hazards assumption, Martingale residuals were calculated and plotted. Stratified Cox models were used if the assumption was violated. Statistical interactions between perceived stress and covariates were tested and none were found. People who retired (*N = 456*), received benefits due to reduced ability to work (*N = 21*), emigrated (*N = 25*) or died (*N = 15*) during follow-up were censored. No loss to follow-up occurred due to the register-based design. Adjusting for lifestyle factors and self-rated health was performed as sensitivity analysis. Furthermore, the association between perceived stress and unemployment with and without preceding sickness absence as outcomes was performed as sensitivity analysis. A sample restricted to those who worked 6 months preceding baseline was also performed as a sensitivity analysis.

Results are presented as hazard ratios (HR) with corresponding 95% confidence intervals (CI). Data management was performed using SAS software, version 9.4 (SAS Institute Inc., Cary, North Carolina, USA). Data analysis was performed using R statistical software package, version 3.3.3 [30].

## Results

**Fig. 2:**
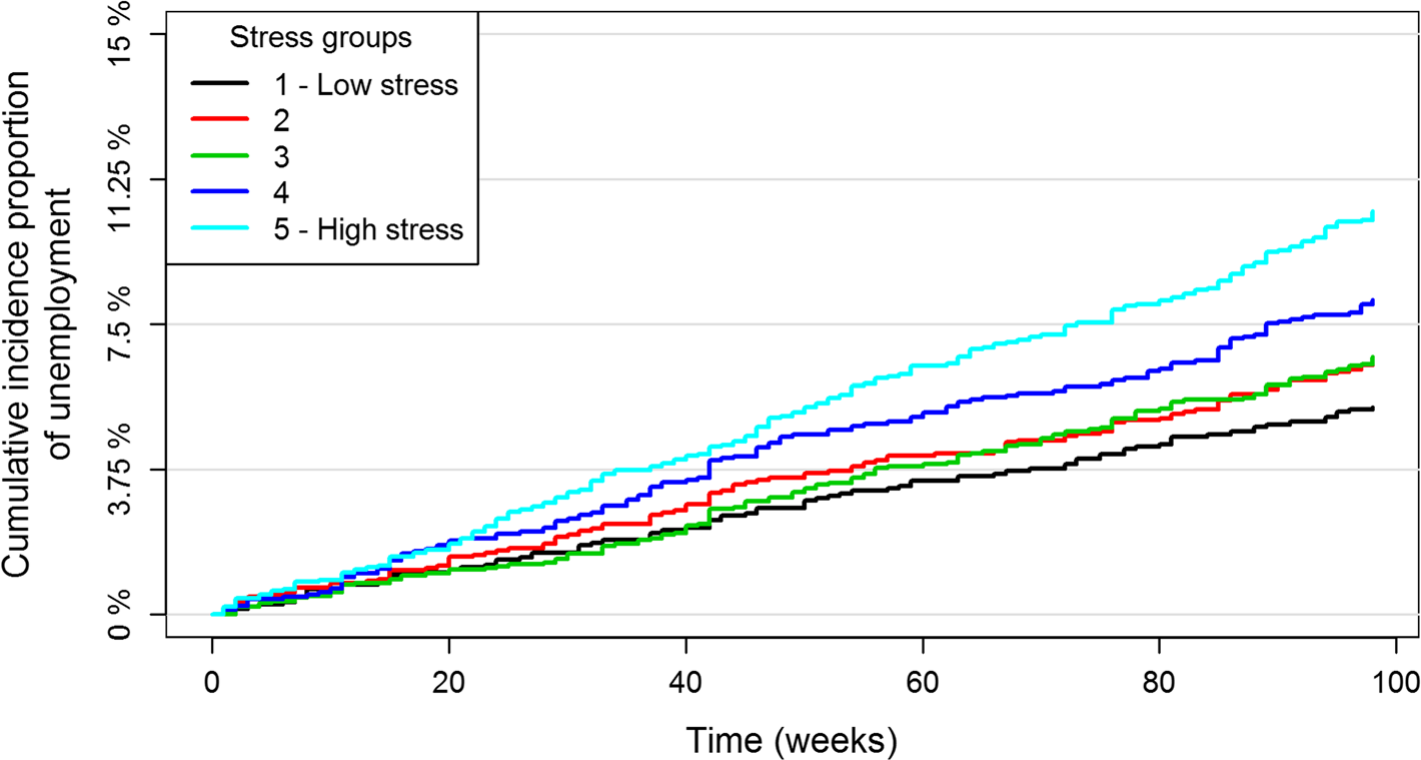
Cumulative incidence proportion of unemployment in different stress quintiles during 98 weeks of follow-up

A high stress level was more common among women, younger age groups and people with a basic education level or low income (Table 1). During follow-up, 224 persons (10.4%) of the high stress group became unemployed, which was higher than the lower stress groups (Fig. 2 and Table 1). There was no association between perceived stress and sickness absence among those who became unemployed during follow-up (*p* = 0.290) (Table 1).

**Table 1:** Baseline characteristics and employment status based on levels of perceived stress (*N = 9335*)

Variables	1 – Low stress (*n* = 1565)^a^	2 (*n* = 1753)^a^	3 (*n* = 2088)^a^	4 (*n* = 1783)^a^	5 - High stress (*n* = 2146)^a^	Total (n = 9335)	*p*-value
Baseline							
Gender							
Women	661 (42.2)	803 (45.8)	1027 (49.2)	884 (49.6)	1192 (55.5)	4567 (48.9)	< 0.01
Age (years)							
18–25	87 (5.6)	101 (5.8)	133 (6.4)	127 (7.1)	189 (8.8)	637 (6.8)	
26–54	1036 (66.2)	1222 (69.7)	1466 (70.2)	1288 (72.2)	1529 (71.2)	6541 (70.1)	
55–64	442 (28.2)	430 (24.5)	489 (23.4)	368 (20.6)	428 (19.9)	2157 (23.1)	< 0.01
Education level^b^							
Basic	359 (22.9)	397 (22.6)	444 (21.3)	424 (23.8)	637 (29.7)	2261 (24.2)	
Vocational	673 (43.0)	744 (42.4)	971 (46.5)	828 (46.4)	961 (44.8)	4177 (44.7)	
Higher	533 (34.1)	612 (34.9)	673 (32.2)	531 (29.8)	548 (25.5)	2897 (31.0)	< 0.01
Household income^c^							
1: < 44,763	324 (20.7)	397 (22.6)	498 (23.9)	453 (25.4)	661 (30.8)	2333 (25.0)	
2: 44,763–55,978	345 (22.0)	428 (24.4)	529 (25.3)	475 (26.6)	558 (26.0)	2335 (25.0)	
3: 55,979–67,524	385 (24.6)	429 (24.5)	538 (25.8)	450 (25.2)	532 (24.8)	2334 (25.0)	
4: > 67,524	511 (32.7)	499 (28.5)	523 (25.0)	405 (22.7)	395 (18.4)	2333 (25.0)	< 0.01
Smoking							
Non smoker	865 (55.6)	908 (52.2)	1095 (52.8)	896 (50.6)	1019 (47.8)	4783 (51.6)	
Former smoker	423 (27.2)	448 (25.7)	554 (26.7)	479 (27.1)	557 (26.1)	2461 (26.5)	
Current smoker	269 (17.3)	385 (22.1)	424 (20.5)	395 (22.3)	556 (26.1)	2029 (21.9)	< 0.01
missing	8	12	15	13	14	62	
Body Mass Index							
Underweight	18 (1.2)	21 (1.2)	17 (0.8)	21 (1.2)	34 (1.6)	111 (1.2)	
Normal weight	699 (45.4)	795 (46.0)	944 (45.7)	799 (45.3)	962 (45.7)	4199 (45.6)	
Overweight	605 (39.3)	668 (38.7)	815 (39.4)	677 (38.4)	742 (35.2)	3507 (38.1)	
Obesity	219 (14.2)	244 (14.1)	290 (14.0)	266 (15.1)	369 (17.5)	1388 (15.1)	0.024
missing	24	25	22	20	39	130	
Alcohol consumption							
Low	1331 (95.1)	1461 (93.2)	1730 (94.2)	1473 (92.9)	1715 (92.4)	7710 (93.5)	
High	69 (4.9)	107 (6.8)	107 (5.8)	113 (7.1)	141 (7.6)	537 (6.5)	0.017
missing	165	185	251	197	290	1088	
Self-rated health							
Good	1538 (98.7)	1715 (98.3)	2039 (97.9)	1692 (95.4)	1882 (88.4)	8866 (95.5)	
Poor	21 (1.3)	29 (1.7)	44 (2.1)	81 (4.6)	247 (11.6)	422 (4.5)	< 0.01
missing	6	9	5	10	17	47	
Outcome:							
Employment status^d^							
Employed	1369 (87.5)	1537 (87.7)	1834 (87.8)	1553 (87.1)	1817 (84.7)	8110 (86.9)	
Censored^e^	112 (7.2)	100 (5.7)	115 (5.5)	85 (4.8)	105 (4.9)	517 (5.5)	
Unemployed	84 (5.4)	116 (6.6)	139 (6.7)	145 (8.1)	224 (10.4)	708 (7.6)	< 0.01
- No sickness absence	78 (92.9)	100 (86.2)	120 (86.3)	130 (89.7)	189 (84.4)	617 (87.1)	
- Sickness absence	6 (7.1)	16 (13.8)	19 (13.7)	15 (10.3)	35 (15.6)	91 (12.9)	0.290

^a^PSS-scores at baseline: 1 – Low stress: 0–5, 2: 6–8, 3: 9–11, 4: 12–14 and 5 – High stress: 15–40

^b^Basic: ISCED level 0–3; early childhood education, primary education, lower secondary education and general upper secondary education. Vocational: ISCED level 3; vocational upper secondary education. Higher: ISCED level 5–8; bachelor’s, master’s and doctor or equivalent level

^c^Divided into quartiles (Euro). Exchange rate: 1 Euro = 7.4396 Danish Kroner, 30th May 2017

^d^Analysis of 18–64-year-old employed non-respondents of the PSS in the North Denmark Health Profile 2010 (*N = 4*809): Employed: 3980 (82.8%). Censored: 262 (5.5%). Unemployed: 567 (11.8%)

^e^People who retired, received benefit due to reduced ability to work, emigrated or died

**Fig. 3:**
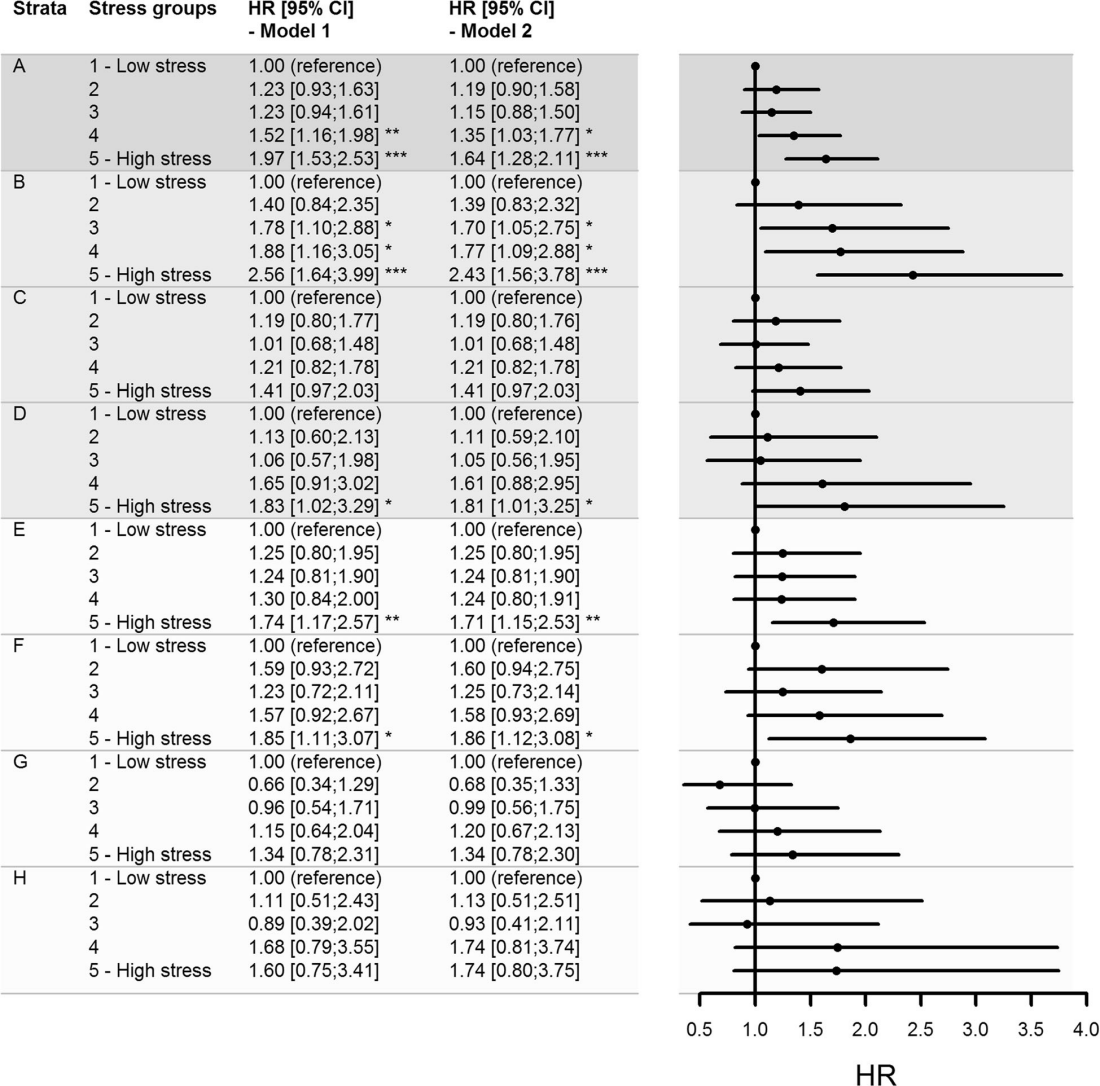
Hazard ratios and 95% confidence intervals of unemployment by stress quintiles. **a** All participants, **b** Basic education level, **c** Vocational level, **d** Higher education level, E: Income quartile 1, **f** Income quartile 2, **g** Income quartile 3 and H: Income quartile 4. Model 1: Unadjusted. Model 2, A: Adjusted for gender, age, education and income level. Model 2, B-H: Adjusted for gender and age. *** *p* < 0.001, ** *p* < 0.01, * *p* < 0.05

The unadjusted and adjusted risks of unemployment by stress quintiles are shown in Fig. 3. After adjusting for gender, age and SES, the risk of unemployment was 1.64 (95% CI: 1.28;2.11) in the high stress group compared to the low stress group. The same trend was observed across education levels after adjusting for gender and age, though with borderline statistical significance among people with vocational or higher education (Fig. 3b-d). Among the lower income groups, a high level of perceived stress increased the risk of unemployment compared to a low level (Fig. 3e + f). In contrast, a higher level of perceived stress did not increase risk of unemployment in the higher income groups (Fig. 3g + h). No statistically significant interaction with the level of perceived stress was found across education levels (*p* = 0.587) or income levels (*p* = 0.841).

### Sensitivity analyses

A high level of perceived stress increased the risk of unemployment with preceding sickness absence (adjusted HR: 3.26 95% CI: 1.35;7.84) and unemployment without preceding sickness absence (adjusted HR: 1.51, 95% CI: 1.16;1.97).

Adjusting for lifestyle factors and self-rated health, showed that the risk of unemployment by perceived stress was similar to the results not adjusted for this (HR: 1.65 95% CI: 1.25;2.19) (*N = 8046*) (Additional file 2: Table S2).

The association between perceived stress and risk of unemployment in the restricted study population was similar for all respondents (adjusted HR: 1.65 95% CI: 1.27;2.14) (*N = 8877*) and SES-groups (Additional file 3: Fig. S1).

## Discussion

This cohort study found that high levels of perceived stress in a working population nearly doubled the risk of subsequent unemployment after adjusting for confounders. No statistically significant interaction by SES was found, suggesting that the risk was present in all SES-groups.

The association between perceived stress and unemployment has previously been investigated primarily through cross-sectional studies [1, 2] in which the direction of causation is unknown. A previous prospective cohort study found that stress increased the risk of subsequent long-term unemployment but not short-term unemployment among 40–59-year-old male construction workers [8]. This was in line with our study, but in contrast to the study by Leino-Arjas et al., we focused on the incidence of unemployment instead of the duration because unemployment itself has been suggested to have adverse consequences [31]. A newly published Danish follow-up study found a higher risk of passive labour market participation, including unemployment, from perceived stress among young women but not among men [32]. Contrary to the study by Trolle et al., we found similar risks of unemployment due to perceived stress among both men and women (Additional file 4: Table S3). However, Trolle et al. [32] did not examine unemployment itself and only a young population was followed, which could blur the comparison with this study.

Psychological distress has also been found to increase the risk of unemployment [33, 34], which supported the finding of this study. However, a meta-analysis found the association between distress and risk of unemployment to be weak [34]. Furthermore, psychological distress only increased the risk of unemployment due to lay-offs but not due to company closings [33], but we were not able to differentiate between reasons for unemployment.

High levels of perceived stress has been found to increase the likelihood of sickness absence [10], which increased the risk of unemployment [3]. The increased risk of unemployment from perceived stress might thus be due to sickness absence preceding unemployment. We investigated this by including sickness absence in a sensitivity analysis, but this was not supported. Besides sickness absence [3], decreased work ability and job satisfaction have been found to increase the risk of unemployment [35] and were affected by perceived occupational stress [36, 37]. Although we measured perceived stress based on the participants’ everyday life and not occupation, PSS has been found to be a suitable measure of occupational stress [38]. Decreased work ability and low job satisfaction might thus be possible explanations for the increased risk of unemployment in this study. Furthermore, job insecurity has also been found to increase perceived stress [39]. A high level of stress could thus be due to upcoming unemployment causing job insecurity. Hereby, the possibility of reverse causality cannot be excluded.

We included both education and income level as indicators of SES as both have been associated with perceived stress [11, 12] and unemployment [5, 14]. We found that the prevalence of high stress was highest among the low SES group consistent with existing literature [11, 12]. We did not find any statistically significant interaction between education or income level and perceived stress and thus no SES differences in the association between perceived stress and risk of unemployment. This might be due to lack of power in the small subgroups.

In spite of the statistically non-significant interaction between perceived stress and education or income, the associations within the education and income subgroups seemed to differ. In all the subgroups of education level, high levels of perceived stress increased the risk of unemployment, though with borderline statistical significance among people with a vocational or higher education. In contrast, high levels of perceived stress only increased the risk of unemployment in the lower income groups. However, the lack of a statistically significant interaction between income and perceived stress remained when income was divided into thirds (*p* = 0.457) or two groups (*p* = 0.376). A possible explanation might be that although low SES-groups have been found to be disadvantaged concerning possible consequences of stress, this cannot always be generalized [13]. Another possible explanation might be that people react and handle stress differently due to different individual and contextual factors [40], which can be difficult to capture in register studies.

### Strengths and limitations

The longitudinal setup separated perceived stress and unemployment in time. We only included 18–64-year-olds who were working at baseline and the preceding 3 months to represent a working population. The register-based design enabled complete follow-up data by which selection during follow-up was prevented. However, the incidence of unemployment was higher among non-respondents compared to respondents (11.8% vs. 7.6%) of the North Denmark Health Profile 2010. Non-respondents were likely to be in the high stress group and selection bias due to non-response might therefore occur. Particularly young men did not respond and had a lower level of perceived stress compared to women [16]. Our results were similar for men and women (Additional file 4: Table S3), but due to the higher risk of unemployment among non-respondents, the results of our analysis might be overestimated.

The comprehensive use of registers limited the risk of information bias compared with studies only using self-reported data [8]. The Income Statistics and Population’s Education Register are of high quality [19, 20]. The DREAM register was feasible for historical register-based follow-up studies of exit from the labour market [18]. Those who did not receive any social transfer payments at baseline and the preceding 3 months were included to represent a working population. However, an unknown number of unemployed individuals might be categorized as working. The two Danish types of unemployment benefits have certain economic and education requirements [23, 24] and some unemployed individuals will not meet the eligibility criteria for neither unemployment insurance benefits nor social insurance benefits. Selection bias only occurs if the possible included unemployed differ concerning both perceived stress and risk of unemployment. Generally, unemployed people experience higher levels of perceived stress compared to those working [1, 2] and a different risk of unemployment. The possible inclusion of unemployed in the employed population might therefore increase the level of perceived stress of the study population at baseline. This would lead to bias towards null with an underestimation of the results. However, the positive predictive value of employment in DREAM has been shown to be more than 90% [18], meaning that 90% of those registered as employed are in fact holding a job. This indicates that the risk of reduced representativeness of the included study population will be of minor importance. The predictive value is lower concerning unemployment benefit, social assistance and sickness benefit [18]. The risk of non-differential misclassification of unemployment might thus be increased. However, the predictive value of positive registration in DREAM was compared to self-reported data and self-reported data on temporary payments like sickness benefits might be biased [18].

Information on stress was assessed with the previously validated PSS, which has been used extensively [41]. We conceptualized the effect of income through household income, which was based on the average from 3 years to get stable values, as income often change over time. The household income was divided by 1.5 when living with a partner to partially allow for sharing of resources when living more people in a household, although this did not allow for children living at home [26].

The included study population was followed for almost 3 years and the level of stress might have changed during follow-up. However, the high stress groups seemed to have the highest cumulative incidence proportion of unemployment throughout follow-up; however unclear at the beginning of follow-up. Cox regression modelling enabled censoring of people who were unlikely to return to the work force or died during follow-up. The censored observations included people with reduced ability to work as they are likely to have a higher level of perceived stress and increased risk of unemployment compared to those remaining under observation. This might bias the observed association between perceived stress and unemployment, but the small size of this group (*N = 21*) limited the potential risk of bias. We included relevant covariates and potential confounders such as age, gender, education and income level. Lifestyle and health factors were not included in the main analyses because they were interpreted as possible intermediate factors. These factors were included in sensitivity analyses showing similar results and did not seem to change the association. Unknown underlying mechanisms might account for the increased risk of unemployment.

This study was based on data from the North Denmark Region in 2010. Around this time many manufacturing companies were hit by large cutbacks and company closures due to the financial crisis. In Denmark, large layoffs were preceded by notice to employees [42]. We only included people employed at baseline and 3 months prior to baseline to exclude recently employed. Despite this, notifications of layoffs could be more than 3 months in cases of cutbacks and company closures and cause perceived job insecurity. Hereby, this unmeasured confounder might upwardly bias the results. However, similar results were found when the study population was restricted to employed people at baseline and preceding 6 months. Furthermore, no statistically significant interactions were found between perceived stress and income or education level. The influence of the layoffs might thus be limited as mainly low income and education groups were affected.

The risk of unemployment might depend on the unemployment rate in specific occupations, but data on occupation was unavailable. The unemployment rate in the society could also influence the risk of unemployment [1]. However, in this study the societal unemployment rate was irrelevant as the participants were followed in the same period. Due to the economic situation in the North Denmark Region, a higher unemployment rate was evident especially among low SES-groups. The higher unemployment rate could decrease the generalizability to the rest of Denmark, other comparable countries and time periods. However, adjustment for SES might partly account for the higher unemployment rate and this did not change the results considerably. The underrepresentation of especially young men might though decrease the generalizability to this group. In contrast to previous studies [8, 32], we included participants, independently of their occupation and gender and covered a wide age range, which improved the generalizability.

## Conclusions

In conclusion, high levels of perceived stress increased the risk of subsequent unemployment. Unemployment has been stated as a public health problem and thus an important topic of prevention. This study contributes with information of perceived everyday life stress as a risk factor of unemployment. No statistically significant interactions between perceived stress and SES were found. This indicates that stress prevention among the working population should not solely focus on stress in the workplace but also include stress from everyday life.

## Additional Files

Additional file 1: **Table S1.** Groupings based on code and corresponding transfer payment in the Danish Register for Evaluation of Marginalization (DREAM). (DOCX 16 kb)

Additional file 2: **Table S2.** Hazard ratios (HR) and 95% confidence intervals (CI) of unemployment by perceived everyday life stress quintiles. Unadjusted (model 1) and adjusted for gender, age, education level, income level, smoking, BMI, alcohol consumption and self-rated health (model 2). Complete cases (*N = 8046*). (DOCX 14 kb)

Additional file 3: **Figure S1.** Hazard ratios and 95% confidence intervals of unemployment by stress quintiles restricted to those who worked 6 months preceding baseline (*N* = 8877). A: All participants, B: Basic education level, C: Vocational level, D: Higher education level, E: Income quartile 1, F: Income quartile 2, G: Income quartile 3 and H: Income quartile 4. Model 1: Unadjusted. Model 2, A: Adjusted for gender, age, education and income level. Model 2, B-H: Adjusted for gender and age. *** *p* < 0.001, ** *p* < 0.01, * *p* < 0.05. (PNG 94 kb)

Additional file 4: **Table S3.** Hazard ratios (HR) and 95% confidence intervals (CI) of unemployment by perceived everyday life stress quintiles among men (*N* = 4567) and women (*N* = 4768). Unadjusted (model 1) and adjusted for age, education and income level (model 2). (DOCX 18 kb)

## Abbreviation

SES: Socioeconomic status
