# Supplementary material for: Perceived stress as a risk factor of unemployment: a register-based cohort study
Source: BMC Public Health. 2018 Jun 13;18:728. doi: 10.1186/s12889-018-5618-z (PMC5998595; doi:10.1186/s12889-018-5618-z)
Supplement: Supplementary file 1 — Table S1. Groupings based on code and corresponding transfer payment in the Danish Register for Evaluation of Marginalization (DREAM). (DOCX 16 kb) [file 12889_2018_5618_MOESM1_ESM.docx]

**Additional file 1**

Table S1: Groupings based on code and corresponding transfer payment in the Danish Register for Evaluation of Marginalization (DREAM).

| **Codes** | **Transfer payments** | **Primary grouping** | **Secondary grouping** | **Follow-up** |
| --- | --- | --- | --- | --- |
| No entry^a^  121-123^a^  124-126 | No transfer payment  Holiday allowance, from employment  Holiday allowance, from unemployment | Employed | Working population | Right-censored (0) |
| 720-29  730-739 | Social security benefit, non-available for work^b^, < 30 years old and no education  Social security benefit, non-available for work^b^ | Unemployed, temporary non-available for work |  |  |
| 412, 413 | Leave of absence | Leave of absence, temporary non-available for work |  |  |
| 881 | Maternity leave pay |  |  |  |
| 651, 652  661 | State education fund grants | Students, temporary non-available for work |  |  |
| 521 | Adult-apprentice | Students, temporary non-available for work |  |  |
| 890-899 | Sickness benefit | Sickness benefit, temporary non-available for work |  | Right-censored (0)^b^ |
| 111-113  130-139  140-149  151-152  211-299 | Voluntary unemployment insurance benefit, full- and part-time  Social security benefit, ready to undertake work  Social security benefit, ready to undertake work, < 30 years old and no education  Voluntary unemployment insurance benefit, extended  Voluntary unemployment insurance benefit, while in job training | Unemployed, ready to undertake work | Unemployed | Event of interest (1) |
| 621  998 | Voluntary early retirement  Public retirement pension | Retired | Retirement | Censored (2) |
| 750-768 | Vocational rehabilitation benefit | Vocational rehabilitation | Reduced ability to work |  |
| 771-774  740-748 | Flexjob^c^  Unemployed after flexjob^c^ | Flexjob |  |  |
| 781-784  783 | Light job (job for disability pensioners)  Disability pension | Disability pension |  |  |
| 997 | Emigrated | Censored |  |  |
|  | Dead^d^ | Censored |  |  |

^a^Inclusion criteria

^b^Due to sickness, maternity leave etc.

Included as part of outcome in analyses concerning perceived stress and risk of unemployment with or without preceding sickness absence.

^c^Job for people with reduced ability to work.

^d^Information from the Danish Register of Causes of Death.
