# Supplementary material for: Perceived stress as a risk factor of unemployment: a register-based cohort study
Source: BMC Public Health. 2018 Jun 13;18:728. doi: 10.1186/s12889-018-5618-z (PMC5998595; doi:10.1186/s12889-018-5618-z)
Supplement: Supplementary file 2 — Table S2. Hazard ratios (HR) and 95% confidence intervals (CI) of unemployment by perceived everyday life stress quintiles. Unadjusted (model 1) and adjusted for gender, age, education level, income level, smoking, BMI, alcohol consumption and self-rated health (model 2). Complete cases (N = 8046). (DOCX 14 kb) [file 12889_2018_5618_MOESM2_ESM.docx]

**Additional file 2**

Table S2: Hazard ratios (HR) and 95% confidence intervals (CI) of unemployment by perceived stress quintiles. Unadjusted (model 1) and adjusted for gender, age, education level, income level, smoking, BMI, alcohol consumption and self-rated health (model 2). Complete cases (*N=8,046*).

| Stress groups |  |  | HR 95% CI  Model 1 | HR 95% CI  Model 2 |
| --- | --- | --- | --- | --- |
| 1 - Low stress |  |  | 1.00 (reference) | 1.00 (reference) |
| 2 |  |  | 1.35 [0.99;1.83] | 1.30 [0.95;1.76] |
| 3 |  |  | 1.19 [0.88;1.61] | 1.08 [0.80;1.45] |
| 4 |  |  | 1.51 [1.12;2.03]** | 1.31 [0.97;1.77] |
| 5 - High stress |  |  | 2.02 [1.53;2.67]*** | 1.67 [1.26;2.21]*** |

*** p<0.001, ** p<0.01, * p<0.05
