# Supplementary figures and images for: Perceived stress as a risk factor of unemployment: a register-based cohort study
Source: BMC Public Health. 2018 Jun 13;18:728. doi: 10.1186/s12889-018-5618-z (PMC5998595; doi:10.1186/s12889-018-5618-z)

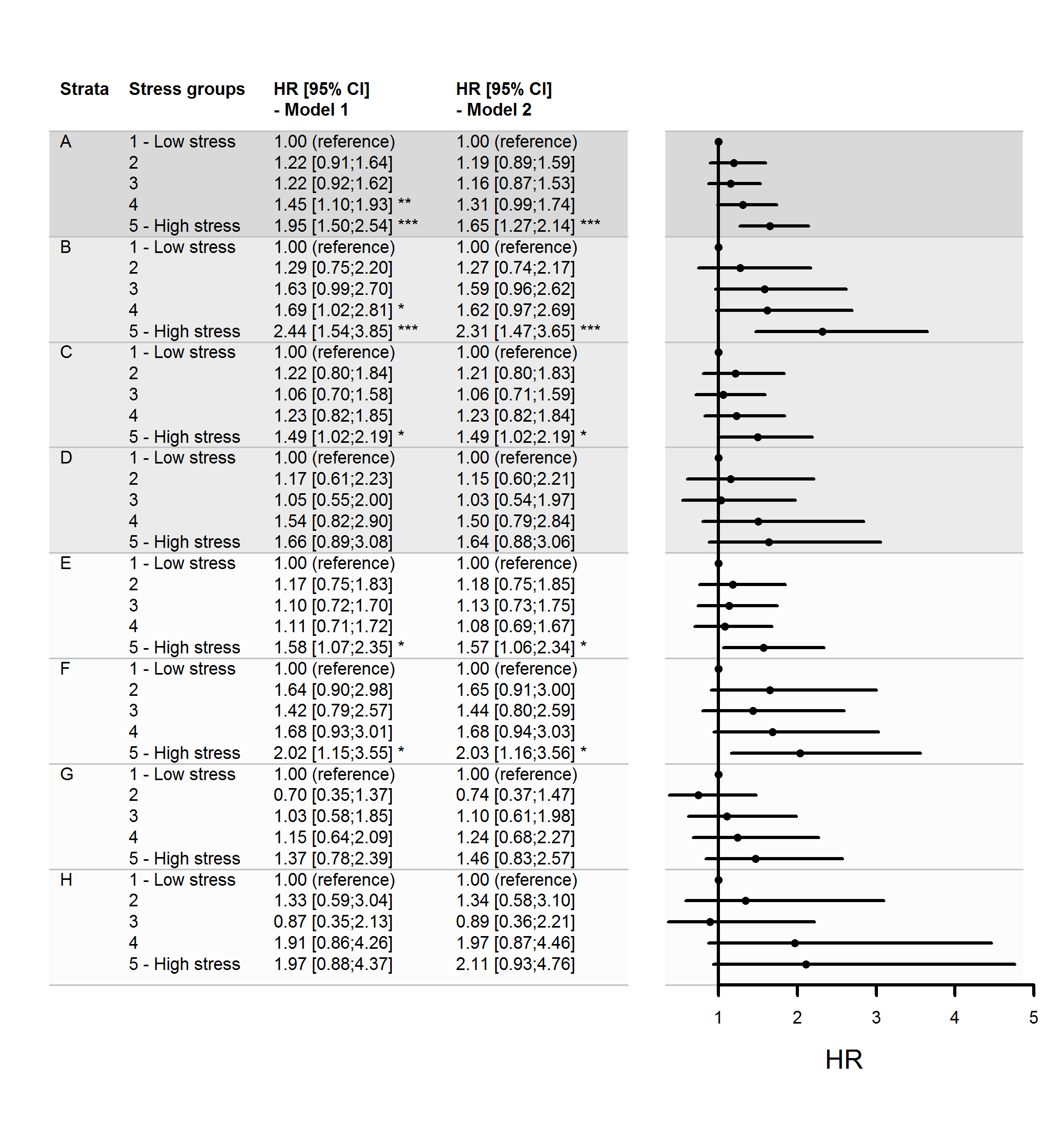

Supplement: Supplementary file 3 — Figure S1. Hazard ratios and 95% confidence intervals of unemployment by stress quintiles restricted to those who worked 6 months preceding baseline (N = 8877). A: All participants, B: Basic education level, C: Vocational level, D: Higher education level, E: Income quartile 1, F: Income quartile 2, G: Income quartile 3 and H: Income quartile 4. Model 1: Unadjusted. Model 2, A: Adjusted for gender, age, education and income level. Model 2, B-H: Adjusted for gender and age. *** p < 0.001, ** p < 0.01, * p < 0.05. (PNG 94 kb) [file 12889_2018_5618_MOESM3_ESM.png]
