# Supplementary material for: Perceived stress as a risk factor of unemployment: a register-based cohort study
Source: BMC Public Health. 2018 Jun 13;18:728. doi: 10.1186/s12889-018-5618-z (PMC5998595; doi:10.1186/s12889-018-5618-z)
Supplement: Supplementary file 4 — Table S3. Hazard ratios (HR) and 95% confidence intervals (CI) of unemployment by perceived everyday life stress quintiles among men (N = 4567) and women (N = 4768). Unadjusted (model 1) and adjusted for age, education and income level (model 2). (DOCX 18 kb) [file 12889_2018_5618_MOESM4_ESM.docx]

**Additional file 4**

Table S3: Hazard ratios (HR) and 95% confidence intervals (CI) of unemployment by perceived stress quintiles among men (*N=4,567*) and women (*N=4,768*). Unadjusted (model 1) and adjusted for age, education and income level (model 2).

| Women (n= 4567, number of events= 315) | | | | |
| --- | --- | --- | --- | --- |
| Stress groups |  |  | HR 95% CI  Model 1 | HR 95% CI  Model 2 |
| 1 - Low stress |  |  | 1.00 (reference) | 1.00 (reference) |
| 2 |  |  | 0.97 [0.59;1.58] | 0.91 [0.55;1.49] |
| 3 |  |  | 1.48 [0.96;2.29] | 1.36 [0.88;2.11] |
| 4 |  |  | 1.65 [1.06;2.56]* | 1.44 [0.93;2.25] |
| 5 - High stress |  |  | 2.37 [1.58;3.55]*** | 1.91 [1.27;2.88]** |
| Men (n= 4768, number of events= 393) | | | | |
| 1 - Low stress |  |  | 1.00 (reference) | 1.00 (reference) |
| 2 |  |  | 1.42 [1.01;2.00]* | 1.40 [0.99;1.99] |
| 3 |  |  | 1.11 [0.78;1.57] | 1.02 [0.72;1.45] |
| 4 |  |  | 1.49 [1.06;2.10]* | 1.33 [0.94;1.88] |
| 5 - High stress |  |  | 1.78 [1.28;2.47]*** | 1.48 [1.07;2.05]* |

*** p<0.001, ** p<0.01, * p<0.05
